# Peer victimization, depression, and non-suicidal self-injury among Chinese adolescents: the moderating role of the *5-HTR2A* gene rs6313 polymorphism

**DOI:** 10.1186/s13034-022-00532-4

**Published:** 2022-12-27

**Authors:** Meijin Li, Huahua Wang, Jingjing Li, Yuting Deng, Chengfu Yu

**Affiliations:** 1 Department of Psychology and Research Center of Adolescent Psychology and Behavior, School of Education, Guangzhou University, Guangzhou, 510006 China; 2 School of Psychology, South China Normal University, Guangzhou, 510631 China

**Keywords:** Peer victimization, Depression, Non-suicidal self-injury, The *5-HTR2A* gene rs6313 polymorphism, Adolescent

## Abstract

**Background:**

Peer victimization is a crucial risk predictor for adolescent non-suicidal self-injury (NSSI). However, adolescent NSSI reactions to peer victimization exhibit large individual differences. This study explored whether depression mediated the association between peer victimization and adolescent NSSI, and whether this mediating path was moderated by the *5-HTR2A* gene rs6313 polymorphism.

**Methods:**

A total of 667 adolescents (*Mean*_age_ = 12.81, *SD* = 0.48) completed questionnaires regarding peer victimization, depression, and NSSI. Genomic DNA was extracted from saliva and buccal cells from each participant.

**Results:**

The results showed that the positive relation between peer victimization and adolescent NSSI was mediated by depression. Moreover, the triple interaction between peer victimization, rs6313 polymorphism, and gender on adolescent depression was significant. And the triple interaction between depression, rs6313 polymorphism, and gender on adolescent NSSI was also significant. Specifically, the risk effect of peer victimization on adolescent NSSI through increased depression was stronger for female adolescents with CC genotype than for female adolescents with CT or TT genotype, and male adolescents with CT or TT genotype. However, the indirect effect was nonsignificant for male adolescents with CC genotype.

**Conclusions:**

These findings promote the etiological understanding of adolescent NSSI, highlighting the mediating and moderating effect between peer victimization and NSSI, and adding evidence supporting the relationship between the *5-HTR2A* gene rs6313 polymorphism, depression and adolescent NSSI.

## Background

Non-suicidal self-injury (NSSI) is the direct, deliberate, and socially unacceptable destruction of one’s body tissues without suicidal intent [1]. Adolescence is a highly sensitive period for the emergence of NSSI. Zhu et al. [2] investigated 1987 Chinese adolescents and found that 15% of the participants had engaged in NSSI in the past 6 months. NSSI is a highly predictive factor for future suicidal ideation [3, 4]. Therefore, it is essential to identify mechanisms that place adolescents at risk for NSSI and develop targeted intervention programs.

According to the interpersonal model of NSSI [1], negative personal relationships often lead to NSSI, which is regarded as a coping strategy to relieve the pressure or pain caused by miserable interpersonal experiences. Evidence is accumulating that peer victimization was significantly linked to adolescent NSSI [5–7]. A systematic review utilizing data from twenty-nine studies indicated that peer victimization is more common among teenagers who have engaged in NSSI compared to those without NSSI history [5]. Wang et al. [7] reported that more than 18.9% of the children had been involved in NSSI after being bullied. These findings highlight that peer victimization was a critical hazard predictor for adolescent NSSI.

According to the vulnerability-stress model [8], depression may mediate the relationship between peer victimization and NSSI. First, adolescents who encounter peer victimization are more likely to experience depression. This may be because they experience a sense of being unworthy and disconnected from peers, which can trigger fierce dysfunctional emotional responses and states, such as depression [9]. According to a three-wave longitudinal study of 2381 Chinese adolescents, Wei et al. [10] demonstrated that peer victimization could positively predict depression in the next 6 months.

Second, when adolescents experience a moderate-high level of depression, they are more likely to develop NSSI compared to adolescents who experience a moderate-low level of depression. NSSI is a compensatory behavior and an easy and accessible way to alleviate negative emotions, such as depression [1]. Increasing research shows that depression plays a crucial role in predicting NSSI among adolescents [11, 12]. Tang et al. [13] also emphasized the positively significant link between depression and adolescent NSSI. Based on the abovementioned literature and theory, we propose the following hypothesis:

Hypothesis 1: Depression will mediate the relationship between peer victimization and adolescent NSSI.

Although peer victimization is a crucial hazard predictor for depression and NSSI among adolescents, not all adolescents are equally influenced by peer victimization. According to the differential susceptibility model [14], individuals have different levels of susceptibility to their environments (both beneficial and adverse environments). Abnormalities within the serotonergic system have been mentioned repeatedly in the context of NSSI [15, 16]. The serotonin 2A (*5-HTR2A*) receptor single nucleotide polymorphism (SNP) rs6313 is intensively associated with NSSI [17]. In a sample of 281 Chinese patients with depression, Qiu et al. [18] found that the *5-HTR2A* gene rs6313 polymorphism was significantly associated with suicidal behavior. Studies also indicated that compared with T-carriers, individuals with the CC genotype of *5-HTR2A* gene rs6313 polymorphism are at a higher risk of NSSI and attempted suicide [17]. In the context of the gene-environment interaction, rs6313 polymorphism has been confirmed to interact with environmental changes to predict outcomes associated with depression and suicide [19, 20]. With a cross-sectional study design, Jiang and collaborators found that stressful working environment and rs6313 polymorphism have the significant interactive effect on mental health problems (including depression) in Chinese petroleum workers [19]. Furthermore, Ghasemi et al. [20] reported that individuals with the *5-HTR2A* gene rs6313 CC genotype rather than T-carriers may be more susceptible to stressful life events, which leads to suicide. Thus, adolescents with the rs6313 CC genotype may be more susceptible to stressful and negative environments than those with T-carriers. Additionally, previous studies have found gendered differences in the moderating effect of rs6313 between negative life events and suicide [21, 22]. Wrzosek et al. [22] demonstrated that females with the CC genotype of rs6313 polymorphism was associated with higher suicide risk than for female adolescents with CT or TT genotype, and male adolescents with CT or TT genotype. Therefore, the present study included gender as another moderating variable. Based on the literature mentioned above, we propose our second hypothesis:

Hypothesis 2: The *5-HTR2A* gene rs6313 polymorphism will moderate the  link between peer victimization and adolescent NSSI via depression.

**Fig. 1:**
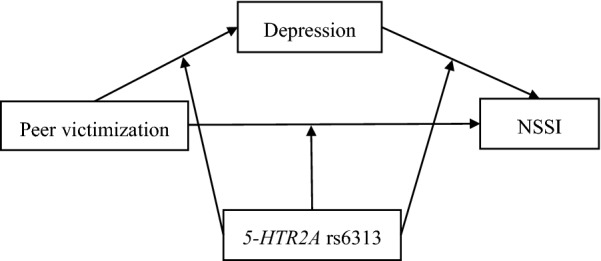
The conceptual model of the proposed moderated mediation framework

The conceptual model is shown in Fig. 1.

## Method

### Participants

A total of 667 junior school students (362 boys and 305 girls) were recruited from Guangdong Province in southern China. The teenagers ranged in age from 12–15 years (*Mean*_age_ = 12.81 years, *SD* = 0.48 years).

### Study design and procedure

This was a cross-sectional study. The cluster sampling method was used to choose subjects and the data were collected in January 2018. Before conducting the research, we acquired the approval from the Ethics in Human Research Committee of the School of Education at Guangzhou University (date of approval: January 15, 2018). After obtaining consent from adolescents and their parents, the self-report questionnaires were distributed and collected by professionally trained postgraduate students in psychology in adolescents' classes. Finally, students were instructed to collect their saliva and give it to the saliva collector. Generally, the questionnaires and saliva collection took 40 to 60 minutes.

### Measures

#### Peer victimization

The Chinese version of the peer victimization questionnaire was adopted to assess peer victimization [23]. This scale contains nine items, which reflect adolescents’ peer victimization experiences (e.g., “Someone is trying to control or bully you”). Adolescents indicated how often they had ever experienced peer victimization during the past 6 months. The scale ranges from 1 (never) to 5 (six or more times). The average score of the 9 items was calculated. Higher scores represented greater peer victimization. The Cronbach’s α of this study was 0.93.

#### Depression

The Center for Epidemiologic Studies Depression Scale [24] was adopted to measure adolescents’ depression. Adolescents were instructed to answer questions about how often their depressive symptoms had occurred during the last week. For example, “I had trouble keeping my mind on what I was doing.” All items were rated on a four-point scale, from 1 (less than 1 day) to 4 (most or all of the time, 5–7 days). We calculated the average score of all items. The higher the score, the greater the degree of depression. The Cronbach’s α of this study was 0.84.

#### NSSI

NSSI was using the Chinese version of the NSSI questionnaire [25]. This questionnaire contains six items. NSSI behaviors included cutting oneself, carving words or patterns on the skin with sharp objects so as to bleed, seriously scratching oneself so as to bleed or leave scars, pulling one’s hair hard, biting oneself, and rubbing one’s skin violently so as to bleed. Adolescents were required to answer how often they engaged in the above-mentioned NSSI behaviors in the past 6 months. A six-point scale ranging from 1 (never) to 6 (several times a week) was used. We calculated the average of these six items. The higher the score, the greater the NSSI. Cronbach’s α was 0.81 in this study.

#### Genotyping

Genomic DNA was extracted from saliva and buccal cells from each participant. In this sample, genotyping yielded three groups as follows: 263 (39.43%) adolescents carried two C alleles (CC), 316 (47.37%) carried one C and one T allele (CT), and 88 (13.19%) carried two T alleles (TT). The *χ*^2^ goodness-of-fit test exhibited that the genotype distribution of the *5-HTR2A* gene rs6313 polymorphism was in keeping with Hardy–Weinberg equilibrium (*χ*^2^ = 0.21, *df* = 2, *p* = 0.65).

#### Control variables

According to existing studies, age and self-esteem have an impact on NSSI [26, 27]; thus, age and self-esteem were included as control variables in our analysis. The scale designed by Rosenberg was adopted to measure the adolescents’ self-esteem [28]. Items included statements such as “I feel I have many good qualities.” All items were scored on a five-point scale ranging from 1 (does not conform at all) to 5 (highly conforms). We calculated the total average score of 10 items. The higher the score, the higher the respondent’s self-esteem. Cronbach’s α was 0.81 in this study.

### Statistical analyses

SPSS 20.0 was used for statistical analyses (SPSS; IBM, Armonk, NY, USA). We adopted Model 4 of PROCESS for SPSS proposed by Hayes [29] to explore whether depression plays a mediating role between peer victimization and adolescent NSSI. To further test the moderating effect of the rs6313 polymorphism and gender on the relationship between peer victimization, depression and adolescent NSSI, Model 73 of PROCESS for SPSS proposed by Hayes [29] was used for data processing.

## Results

### Demographic information

A total of 667 adolescents participated in this study. The mean age of the adolescents was between 12.33 and 13.29 years. Most respondents were male (54%); about 46% were female. Most of the adolescents’ fathers (87.7%) and mothers (89.7%) had lower levels of education, which means that they did not hold bachelor’s, master’s, or doctoral degrees. About half of the respondents’ families (50.4%) had a monthly income of more than 5000 RMB. Additionally, 620 (93%) adolescents were living in two-parent families, 30 (4.5%) were living in single-parent families, and 17 (2.5%) adolescents were living in reconstituted families (see Table 1).

**Table 1:** Demographic characteristics of adolescents (n = 667)

Demographic variables		Values
Age	*Mean*	12.81
Gender n (%)	Female	305 (46%)
	Male	362 (54%)
Father education n (%)	≥ higher education	85 (12.3%)
	< higher education	582 (87.7%)
Mother education n (%)	≥ higher education	69 (10.3%)
	< higher education	598 (89.7%)
Family income n (%)	≥ 5000RMB per month	336 (50.4%)
	< 5000RMB per month	331 (49.6%)
Family structure n (%)	Two-parent families	620 (93%)
	Single-parent families	30 (4.5%)
	Reconstituted family	17 (2.5%)

### Descriptive statistics

A total 16.34% (*n* = 109) of the participants in the current sample displayed NSSI. The means, standard deviations, and correlations of all study variables have been presented in Table 2. Both peer victimization and depression were positively associated with adolescent NSSI, suggesting that peer victimization and depression are risk factors for NSSI. In addition, peer victimization was positively correlated with adolescent depression. However, there were no significant associations between the *5-HTR2A* gene rs6313 polymorphism and peer victimization, depression, and NSSI.

**Table 2:** Descriptive statistics and correlations among all variables

Variable	1	2	3	4	5	6	7
1.Gender	1.00						
2.Age	0.06	1.00					
3.Self-esteem	0.02	−0.03	1.00				
4.Peer victimization	0.12**	−0.03	0.30***	1.00			
5.Depression	−0.06	0.04	−0.57***	0.44***	1.00		
6.NSSI	−0.05	−0.01	−0.13**	0.25***	0.30***	1.00	
7.Gene rs6313	0.01	−0.05	0.01	−0.02	−0.04	−0.03	1.00
*Mean*	–	12.81	1.94	0.30	1.70	1.05	–
*SD*	–	0.48	0.82	0.05	0.43	0.20	–

Gender was coded by male = 1, female = 0

rs6313 polymorphism coded by CC = 0, CT = TT = 1

*p < 0.05, **p < 0.01, ***p < 0.001

### The mediating effect of depression

Table 3 shows the results of the mediation model. After controlling for gender, age, and self-esteem, peer victimization positively predicted depression (*β* = 0.32, *SE* = 0.03, *t* = 10.21, *p* < 0.001, 95% CI [0.26, 0.38]), and depression positively predicted NSSI (*β* = 0.27, *SE* = 0.05, *t* = 5.66, *p* < 0.001, 95% CI [0.18, 0.36]). The residual effect of peer victimization on NSSI was significant (*β* = 0.15, *SE* = 0.04, *t* = 3.63, *p* < 0.001, 95% CI [0.18, 0.36]). The bias-corrected percentile bootstrap method (*n* = 5000 bootstrap samples) revealed that depression had a significant mediating effect on the relationship between peer victimization and NSSI (indirect effect = 0.09, *SE* = 0.03, 95% CI [0.04, 0.15]).

**Table 3:** Testing for the mediation effect of depression

Variable	Equation 1 (Depression)				Equation 2 (NSSI)			
	*β*	*SE*	*t*	95% CI	*β*	*SE*	*t*	95% CI
*Covariates:*								
Gender	−0.183	0.06	−2.91**	[−0.29, −0.06]	−0.11	0.07	−1.47	[−0.08, 0.06]
Age	0.04	0.03	1.49	[−0.01, 0.10]	−0.01	0.04	−0.29	[−0.08, 0.06]
SE	−0.47	0.03	−15.12*****	[−0.53, −0.41]	0.07	0.04	1.57	[−0.17, 0.16]
*Study variables:*								
PV	0.32	0.03	10.21***	[0.26, 0.38]	0.15	0.04	3.63***	[018, 0.36]
Depression	–	–	–	–	0.27	0.05	5.66***	[0.18, 0.36]
*R*^2^	0.42				0.11			
*F*	117.74***				16.82***			

Gender was coded by male = 1, female = 0

*SE* Self-Esteem, *PV* Peer Victimization, *NSSI* Non-Suicidal Self-Injury

*p < 0.05, **p < 0.01, ***p < 0.001

### The moderating effect of the 5-HTR2A gene rs6313 polymorphism

Table 4 shows the results of the moderated mediation model. As shown in Equation 1, the triple interaction between peer victimization, rs6313 polymorphism, and gender on adolescent depression was significant (*β* = 0.38, *SE* = 0.13, *t* = 3.04, *p* < 0.01, 95% CI [0.14, 0.63]). Moreover, as shown in Equation 2, the triple interaction between depression, rs6313 polymorphism, and gender on adolescent NSSI was significant (*β* = 0.38, *SE* = 0.17, *t* = 2.23, *p* < 0.05, 95% CI [0.06, 0.71]). Thus, the indirect effect of “peer victimization → depression → adolescent NSSI” was moderated by rs6313 polymorphism and gender. Specifically, the risk effect of peer victimization on adolescent NSSI through increased depressive symptoms was stronger for female adolescents with CC genotype (indirect effect = 0.28, *SE* = 0.14, 95% CI [0.06, 0.61]) than for female adolescents with CT or TT genotype (indirect effect = 0.03, *SE* = 0.02, 95% CI [0.01, 0.09]), and male adolescents with CT or TT genotype (indirect effect = 0.07, *SE* = 0.02, 95% CI [0.02, 0.12]). However, the indirect effect was nonsignificant for male adolescents with CC genotype (indirect effect = 0.05, *SE* = 0.04, 95% CI [−0.01, 0.16]).

**Table 4:** Testing for the moderating role of the *5-HTR2A* gene rs6313 polymorphism and gender

Variable	Equation 1 (Depression)				Equation 2 (NSSI)			
	*β*	*SE*	*t*	95% CI	*β*	*SE*	*t*	95% CI
*Covariates:*								
Age	0.04	0.03	1.48	[−0.01, 0.10]	−0.01	0.04	-0.20	[−0.08, 0.06]
SE	−0.48	0.03	−15.45	[−0.54, −0.42]	0.06	0.04	1.39	[−0.26, 0.15]
*Study variables:*								
Gender	−0.17	0.06	−2.88**	[−0.29, −0.06]	−0.11	0.07	−1.42	[−0.25, 0.04]
PV	0.32	0.03	10.20***	[0.26, 0.39]	0.16	0.04	3.95***	[0.08, 0.25]
Rs6313	−0.09	0.06	−1.47	[−0.21, 0.03]	−0.03	0.08	−0.36	[−0.18, 0.12]
PV × rs6313	−0.12	0.06	−1.96	[−0.24, 0.00]	0.02	0.09	0.29	[−0.14, 0.19]
PV × Gender	−0.04	0.06	−0.69	[−0.16, 0.08]	−0.11	0.09	−1.26	[−0.28, 0.06]
rs6313 × Gender	0.13	0.12	1.06	[−0.11, 0.37]	0.08	0.15	0.55	[−0.21, 0.38]
PV × rs6313 × Gender	0.38	0.13	3.04**	[0.14, 0.63]	−0.04	0.18	−0.26	[−0.39, 0.30]
Depression	–	–	–	–	0.25	0.05	5.23***	[0.16, 0.35]
Depression × rs6313	–	–	–	–	−0.14	0.08	−1.73	[−0.31, 0.02]
Depression × Gender	–	–	–	–	−0.10	0.08	−1.26	[−0.27, 0.06]
Depression × rs6313 × Gender	–	–	–	–	0.38	0.17	2.30*	[0.06, 0.71]
*R*^2^	0.42				0.14			
*F*	54.47***				7.96***			

Gender was coded by male = 1, female = 0

rs6313 polymorphism coded by CC = 0, CT = TT = 1

*SE* Self-Esteem, *PV* Peer Victimization, *NSSI* Non-Suicidal Self-Injury, *rs6313 5-HTR2A* gene rs6313 polymorphism

* *p* < 0.05, ** *p* < 0.01, ***p < 0.001

## Discussion

Based on the vulnerability-stress model and the differential susceptibility model, this study examined whether depression mediated the relationship between peer victimization and adolescent NSSI, and whether this indirect link was moderated by the *5-HTR2A* gene rs6313 polymorphism.

Consistent with Hypothesis 1, we found that depression significantly mediated the relationship between peer victimization and adolescent NSSI. Social factors, especially peer relationship, seem to play a crucial role in the onset and maintenance of NSSI [30]. When adolescents experience peer victimization, they are more likely to experience depression, which leads to increased NSSI. Therefore, depression is an important, underlying psychosocial mechanism that clarifies that peer victimization is associated with more NSSI. This finding is congruent with the vulnerability-stress model [8], as well as previous research showed that depression can mediate the relation between negative interpersonal relationship (such as peer victimization) and problem behaviors [7, 10, 31]. A previous study [32] specifically examined the influence of peer victimization and depression on NSSI. Unlike this study, our study considers the potential association between peer victimization and depression. Our study enriches the body of research regarding the potential mediating mechanisms between peer victimization and NSSI, which provides empirical evidence that may be helpful when creating intervention projects to decrease the incidence rate of NSSI among adolescents.

Consistent with Hypothesis 2, our study showed that indirect effect of “peer victimization → depression → adolescent NSSI” was moderated by rs6313 polymorphism and gender. Firstly, rs6313 polymorphism and gender moderated the relation between peer victimization and depression directly. Specifically, female adolescents with the CC genotype exhibited more depression when exposed to high levels of peer victimization and less depression when experiencing less peer victimization. Consistent with previous findings, individuals with CC genotype of rs6313 polymorphism may be more susceptible to negative environmental events which may eventually lead to depression [19]. A similar study reported that the moderating effect of serotonin transporter genes between stressful interpersonal event and depression were only significant for female adolescents [33].

Secondly, rs6313 polymorphism and gender moderated the relation between depression and adolescent NSSI. Specifically, compared to female adolescents with CT or TT genotype and all males, female adolescents with CC genotype developed more NSSI when they exhibited depression. Consistent with prior findings, rs6313 polymorphism CC genotype was associated with increased risk of depression and suicide [22, 34]. Another study found that in females, but not in males, those with CC genotype of rs6313 polymorphism had significantly higher level of impulsivity [35]. This may be explain why depressed female adolescents with CC genotype are more prone to develop NSSI. Overall, the indirect association between peer victimization and adolescent NSSI via depression was moderated by the *5-HTR2A* gene rs6313 polymorphism and gender. When CC genotype females experience peer victimization, they will report higher levels of depression than CT or TT genotype and all males, which eventually increases the risk of NSSI. This coincides with previous studies showing that females with CC genotype, but not T-carriers, reported more self-harm behaviors [17] and suicide when experiencing stressful events [20]. It is noteworthy that when female adolescents with the CC genotype have not experienced peer victimization, they will develop less depression, which reduces the occurrence of NSSI. These findings coincide with the differential susceptibility model [14], which highlights that genetic sensitivity to environmental experiences results in increased benefits owing to favorable social contexts but more detriment owing to adverse social contexts. This may explain why those females carrying the CC genotype scored highest on NSSI if they experienced a higher level of peer victimization but lowest if they had grown up with a lower level of peer victimization.

Recently, a study about the neural correlates of peer victimization has demonstrated that experiencing peer victimization would trigger depression in adolescents through increased amygdala responses [36]. Furthermore, a highly expressed *5-HTR2A* genotype (CC) is related to the increased activity of the amygdala and hypothalamic–pituitary–adrenal (HPA) axis [37, 38]. Thus, negative peer relationships and rs6313 polymorphism CC genotype may increase the susceptibility to depression through their synergistic effects on amygdala circuitry. Moreover, epigenetic changes to *5-HTR2A* expression via environmental stressors would be expected to affect brain development and, consequently, behavior [39]. Therefore, epigenetic changes to rs6313 polymorphism via peer victimization may influence brain development (such as HPA axis and amygdala), and subsequently NSSI occurs. Research also demonstrated that the C allele of *5-HTR2A* rs6313 is a risk factor associated with many mental disorders such as depression, NSSI, and suicidal behavior [17, 40]. Enriched previous research [41], the current study found that rs6313 polymorphism and gender moderate the indirect effect between peer victimization and NSSI through increased depression. However, our sample size was small. Further studies are warranted to clarify and confirm the function of the rs6313 polymorphism and gender in modulating the positive indirect association between peer victimization and adolescent NSSI.

Concerning the gender differences in the moderating effect of the rs6313 polymorphism, one possible explanation is that the prevalence rates of NSSI were higher among female adolescents than among male adolescents [42]. Ben-Efraim et al. [21] found that the rs6313 polymorphism moderates the association between negative life events and suicidal behavior in female adolescents. Another research also demonstrated that young adult with the CC genotype of rs6313 was associated with higher suicide risk in females, but not in males [22]. Therefore, we conclude that the effect of gene-environment interaction on NSSI is gender specific.

Several limitations should be considered. First, this study adopted a cross-sectional design and self-reported assessments; thus, the causality between variables cannot be inferred. Future research should consider using a longitudinal design and rigorous and objective measurements to further verify the relationship between peer victimization and NSSI. Second, the study only considers a single gene SNP, which may lead to false positive results [43]. Multiple genes and more variations of *5-HTR2A* should be examined to obtain more information. Third, this study only tested traditional peer victimization; we did not investigate cyberbullying. Several studies showed that cyberbullying seriously affects adolescents’ physical and mental health, resulting in adolescent NSSI [44]. Therefore, future research should include cyberbullying to comprehensively reveal the role of peer victimization on adolescent NSSI and its unique impact mechanism.

Although there are some limitations, the current study has implications for educational researchers and clinical practitioners. Firstly, peer victimization is always a crucial risk predictor for adolescent NSSI. In order to reduce the incidence of NSSI, some prevention intervention measures should be taken, such as teaching adolescents more skills of communication with peers, so that a healthy and harmonious interpersonal communication can be achieved. And it’s necessary to provide available social support to disadvantaged adolescents [45]. Secondly, research showed that peer victimization could increase adolescents’ NSSI via increasing their depression. Thus, adolescents can relieve depression through emotion regulation strategies, which in turn reduce NSSI [46]. Thirdly, individuals vary in their susceptibility to peer environmental influences. We found that female adolescents with rs6313 polymorphism CC genotype female were  more likely to occur emotional dysregulation and NSSI when they experienced peer victimization. Therefore, identifying susceptible individuals is beneficial for clinical practitioners to implement more effective interventions.

## Data Availability

The data presented in this study are available on request from the corresponding authors CY.

## Abbreviations

NSSI: Non-Suicidal Self-Injury
*5-HTR2A*: 5-Hydroxytryptamine Receptor 2A
PV: Peer Victimization

## References

[1] Nock MK (2010). Self-injury. Annu Rev Clin Psychol.

[2] Zhu J, Chen Y, Su B, Zhang W (2021). Anxiety symptoms mediates the influence of cybervictimization on adolescent non-suicidal self-injury: the moderating effect of self-control. J Affect Disord.

[3] Robinson K, Garisch JA, Wilson MS (2021). Nonsuicidal self-injury thoughts and behavioural characteristics: associations with suicidal thoughts and behaviours among community adolescents. J Affect Disord.

[4] Liu S, You J, Ying J, Li X (2020). Emotion reactivity, nonsuicidal self-injury, and regulatory emotional self-efficacy: a moderated mediation model of suicide ideation. J Affect Disord.

[5] Serafini G, Canepa G, Aguglia A, Amerio A (2021). Bullying victimization/perpetration and non-suicidal self-injury: a systematic review. Eur Psychiat.

[6] Victor SE, Hipwell AE, Stepp SD, Scott LN (2019). Parent and peer relationships as longitudinal predictors of adolescent non-suicidal self-injury onset. Child Adolesc Psychiatry Ment Health.

[7] Wang Q, Liu X (2019). Peer victimization, depressive symptoms and non-suicidal self-injury behavior in Chinese migrant children: the roles of gender and stressful life events. Psychol Res Behav Manag.

[8] Hankin BL, Abela JR (2005). Development of psychopathology: a vulnerability-stress perspective.

[9] Macalli M, Orri M, Tzourio C, Côté SM (2021). Contributions of childhood peer victimization and/or maltreatment to young adult anxiety, depression, and suicidality: a cross-sectional study. BMC Psychiatry.

[10] Wei Y, Ren P, Qin X, Zhang Y, Luo F (2022). Adolescent peer victimization and deliberate self-harm: a three-wave moderated mediation model. J Interpers Violence.

[11] Kang L, Li R, Liu H, Ma S (2021). Nonsuicidal self-injury in undergraduate students with major depressive disorder: the role of psychosocial factors. J Affect Disord.

[12] Ghinea D, Fuchs A, Parzer P, Koenig J, Resch F, Kaess M (2021). Psychosocial functioning in adolescents with non-suicidal self-injury: the roles of childhood maltreatment, borderline personality disorder and depression. Borderline Pers Disord Emot Dysregul.

[13] Tang WC, Lin MP, Wu JYW, Lee YT (2022). Mediating role of depression in the association between alexithymia and nonsuicidal self-injury in a representative sample of adolescents in Taiwan. Child Adolesc Psychiatry Ment Health.

[14] Belsky J, Pluess M (2009). Beyond diathesis stress: differential susceptibility to environmental influences. Psychol Bull.

[15] Halicka J, Szewczuk-Bogusawska M, Adamska A, Misiak B (2020). Neurobiology of the association between non-suicidal self-injury, suicidal behavior and emotional intelligence: a review. Arch Psychiatr Psychother.

[16] Desmyter S, Van Heeringen C, Audenaert K (2011). Structural and functional neuroimaging studies of the suicidal brain. Prog Neuro-Psychopharmacol Biol Psychiatry.

[17] Xu X, Zeng Q, Cao J, Chen X (2021). Associations between self-harm behavior and psychosocial-polymorphism in female freshmen. J Shandong University (Health Sci).

[18] Qiu XH, Ma JS, Song JY, Wang L (2015). Association between serotonin receptor genes and suicide behavior. Chin J Public Health.

[19] Jiang T, Ge H, Sun J, Li R (2017). Relationship between occupational stress, 5-HT2A receptor polymorphisms and mental health in petroleum workers in the Xinjiang Arid desert: a cross-sectional study. Int J Environ Res Public Health.

[20] Ghasemi A, Seifi M, Baybordi F, Danaei N (2018). Association between serotonin 2A receptor genetic variations, stressful life events and suicide. Gene.

[21] Ben-Efraim YJ, Wasserman D, Wasserman J, Sokolowski M (2013). Family-based study of HTR2A in suicide attempts: observed gene, gene×environment and parent-of-origin associations. Mol Psychiatr.

[22] Wrzosek M, Łukaszkiewicz J, Wrzosek M, Serafin P (2011). Association of polymorphisms in HTR2A, HTR1A and TPH2 genes with suicide attempts in alcohol dependence: a preliminary report. Psychiatry Res.

[23] Zhou S, Yu C, Xu Q, Wei C (2014). Peer victimization and problematic online game use among junior middle school students: mediation and moderation effects. Educ Meas Eval.

[24] Radloff LS (1977). A self-report depression scale for research in the general population. Appl psychol Meas.

[25] Yu CF, Li MJ, Zhang W (2021). Childhood trauma, parent-child conflict, and adolescent non-suicidal self-injury: the moderating role of OXTR gene rs53576 polymorphism. J Chin Youth Soc Sci.

[26] Muehlenkamp JJ, Xhunga N, Brausch AM (2018). Self-injury age of onset: a risk factor for NSSI severity and suicidal behavior. Arch Suicide Res.

[27] Forrester RL, Slater H, Jomar K, Mitzman S (2017). Self-esteem and non-suicidal self-injury in adulthood: a systematic review. J Affect Disord.

[28] Rosenberg M (1965). Society and the adolescent child.

[29] Hayes, A. F. Model Templates for PROCESS for SPSS and SAS. 2013. pp.61.

[30] Brown RC, Witt A. Social factors associated with non-suicidal self-injury (NSSI). Child Adolesc Psychiatry Ment Health. 2019;13:23–25. 10.1186/s13034-019-0284-1. 10.1186/s13034-019-0284-1 PMC6563365 31210782

[31] Liu J, Liu X, Wang H, Gao Y. Harsh parenting and non-suicidal self-injury in adolescence: the mediating effect of depressive symptoms and the moderating effect of the COMT Val158Met polymorphism. Child Adolesc Psychiatry Ment Health. 2021;15:70–79. 10.1186/s13034-021-00423-0. 10.1186/s13034-021-00423-0 PMC8611980 34814943

[32] Baiden P, Stewart SL, Fallon B (2017). The mediating effect of depressive symptoms on the relationship between bullying victimization and non-suicidal self-injury among adolescents: findings from community and inpatient mental health settings in Ontario. Canada Psychiatry Res.

[33] Constance, Hammen, Patricia, A, Brennan, et al. Chronic and acute stress, gender, and serotonin transporter gene–environment interactions predicting depression symptoms in youth. J Child Psychol Psychiatry. 2010;51(2):180–87. 10.1111/j.1469-7610.2009.02177.x. 10.1111/j.1469-7610.2009.02177.x PMC2883453 19811586

[34] Holmes C, Arranz M, Collier D, Powell J, et al. Depression in Alzheimer's disease: the effect of serotonin receptor gene variation. Am J Med Genet. 2003;119B:40–43. 10.1002/ajmg.b.10068. 10.1002/ajmg.b.10068 12707936

[35] Stoltenberg Scott F., Christ Christa C., Highland Krista B. (2012). Serotonin system gene polymorphisms are associated with impulsivity in a context dependent manner. Progress in Neuro-Psychopharmacology and Biological Psychiatry.

[36] McIver T. Examining the neural correlates of peer victimization and peer defending using functional magnetic resonance imaging (Doctoral dissertation). 2022.

[37] Carneiro-Nascimento S, Powell W, Uebel M, Buerge M (2021). Region-and receptor-specific effects of chronic social stress on the central serotonergic system in mice. IBRO Neurosci Rep.

[38] Petit AC, Quesseveur G, Gressier F, Colle R (2014). Converging translational evidence for the involvement of the serotonin 2A receptor gene in major depressive disorder. Prog Neuro-Psychopharmacol Biol Psychiatry.

[39] Parade SH, Novick AM, Parent J, Seifer R (2017). Stress exposure and psychopathology alter methylation of the serotonin receptor 2A (HTR2A) gene in preschoolers. Dev Psychopathol.

[40] Serretti A, Drago A, De Ronchi D (2007). HTR2A gene variants and psychiatric disorders: a review of current literature and selection of SNPs for future studies. Curr Med Chem.

[41] Arias B, Gastó C, Catalán R, Gutiérrez B (2001). The 5-HT2A receptor gene 102T/C polymorphism is associated with suicidal behavior in depressed patients. Am J Med Genet.

[42] Xin M, Yang X, Liu K, Naz Boke B (2020). Impact of negative life events and social support on nonsuicidal self-injury among Chinese middle school students. Am J Mens Health.

[43] Dick DM, Agrawal A, Keller MC, Adkins A (2015). Candidate gene-environment interaction research: reflections and recommendations. Perspect Psychol Sci.

[44] Faura-Garcia J, Orue I, Calvete E (2021). Cyberbullying victimization and nonsuicidal self-injury in adolescents: The role of maladaptive schemas and dispositional mindfulness. Child Abuse Negl.

[45] Wang Q, Liu X (2021). Peer victimization and nonsuicidal self-injury among Chinese left-behind children: The moderating roles of subjective socioeconomic status and social support. J Interpers Violence.

[46] Sloan E, Hall K, Moulding R, Bryce S, Mildred H, Staiger PK (2017). Emotion regulation as a transdiagnostic treatment construct across anxiety, depression, substance, eating and borderline personality disorders: A systematic review. Clin Psychol Rev.

